# Impact of protease inhibitors on the evolution of urinary markers: Subanalyses from an observational cross-sectional study

**DOI:** 10.1097/MD.0000000000004507

**Published:** 2016-08-12

**Authors:** Anna Bonjoch, Jordi Puig, Nuria Pérez-Alvarez, Javier Juega, Patricia Echeverría, Bonaventura Clotet, Ramón Romero, J. Bonet, E. Negredo

**Affiliations:** a Unitat VIH, Fundació Lluita contra la SIDA, Hospital Germans Trias i Pujol, Universitat Autònoma de Barcelona, Badalona, Spain; b Statistics and Operations Research Department, Universitat Politècnica de Catalunya, Spain; c Nefrology department, Hospital Germans Trias i Pujol, Spain; d IrsiCaixa Foundation, Spain; e Universitat de Vic-Universitat Central de Catalunya, Barcelona, Spain.

**Keywords:** AIDS, HIV, kidney impairment, nephrotoxicity, protease inhibitors, proteinuria

## Abstract

Kidney injury (defined as the presence of albuminuria, proteinuria, glycosuria [without hyperglycemia], hematuria, and/or renal hypophosphatemia) is an emerging problem in human immunodeficiency virus (HIV)-infected patients, although few data are available on the role of protease inhibitors (PIs) in this condition.

To determine the time to kidney injury in a cohort of HIV-infected patients receiving a PI-containing regimen.

We report the results of a subanalysis of a published cross-sectional study. The subanalysis included only patients receiving PI-containing regimens for more than 6 months (377 of the overall 970 patients). We determined associated factors and constructed receiver operating characteristic curves to estimate time to kidney injury depending on the PI used.

The percentage of patients with kidney injury was 27.7% for darunavir, 27.9% for lopinavir, and 30% for atazanavir. Time to kidney injury was as follows: 229 days for atazanavir/ritonavir (area under the curve [AUC], 0.639; sensitivity, 0.89; specificity, 0.41); 332 days for atazanavir/ritonavir plus tenofovir (AUC, 0.603; sensitivity, 0.75; and specificity, 0.29); 318 days for nonboosted atazanavir (AUC, 0.581; sensitivity, 0.89; and specificity, 0.29); 478 days for lopinavir/ritonavir (AUC, 0.566; sensitivity, 0.864; and specificity, 0.44); 1339 days for lopinavir/ritonavir plus tenofovir (AUC, 0.667; sensitivity, 0.86; and specificity, 0.77); 283 days for darunavir/ritonavir (AUC, 0.523; sensitivity, 0.80; and specificity, 0.261); and 286 days for darunavir/ritonavir plus tenofovir (AUC, 0.446; sensitivity, 0.789; and specificity, 0.245). The use of lopinavir/ritonavir without tenofovir was a protective factor (odds ratio = 1.772; 95%CI, 1.070–2.93; *P* = 0.026).

For all PIs, the percentage of patients with kidney injury exceeded 27%, irrespective of tenofovir use. The longest time to kidney injury was recorded with lopinavir/ritonavir. These results demonstrate the need for renal monitoring, including urine samples, in patients receiving a PI-based regimen, even when tenofovir is not used concomitantly.

## 1 Introduction

Renal abnormalities (defined as alteration in urine and blood markers, i.e., presence of microalbuminuria, macroalbuminuria, or proteinuria and/or the presence of glycosuria [without hyperglycemia], hematuria, and/or hypophosphatemia, or an estimated glomerular filtration rate [eGFR] < 60 mL/min/1.73 m^2^) are an emerging problem in the human immunodeficiency virus (HIV)-infected population owing to increasing life expectancy and prolonged exposure to antiretroviral drugs. Prevalence is as high as 30% in some series. ^[1]^ Kidney abnormalities usually develop slowly and silently, but can progress to irreversible chronic disease. However, the most relevant consequence of some alterations such as proteinuria or altered GFR is probably the increased risk of cardiovascular events and death.[ ^2^ ^3^]

The relationship between antiretroviral agents and renal abnormalities has been established mainly for tenofovir disoproxil fumarate (TDF) and for its association with tubular toxicity. TDF-related kidney injury is more likely when patients receive concomitant protease inhibitors (PIs) owing to pharmacokinetic interactions.[ ^4^ ^5^] One meta-analysis pointed to a decline in kidney function associated with PIs, independently of concomitant use of tenofovir. ^[6]^ Nonetheless, few data are available on the impact of PIs on proteinuria and other signs of kidney injury.

The aim of the present study was to determine the role of PIs in kidney injury by evaluating time to appearance of renal abnormalities in a cohort of HIV-infected patients receiving a PI-containing regimen.

## 2 Methods

We performed a subanalysis of data collected from a cross-sectional study. ^[7]^ Briefly, the main study included 970 HIV-infected outpatients who consecutively attended our HIV Care Unit and agreed to participate (from January 2011 to December 2012). All the patients signed an informed consent document, and the Institutional Ethics Committee of the hospital and the local health authorities approved the study (code: EO-10-035).

For the subanalysis, we selected those patients who were receiving a PI-containing regimen for more than 6 months and had an eGFR > 60 mL/min/1.73 m^2^ (377 out of 970). The exclusion criteria included baseline renal disease, any baseline renal alterations (baseline defined as the time of the initiation of the current antiretroviral regimen), and extra renal causes of glycosuria and hypophosphatemia. The objective was to compare the impact of the 3 most commonly used PIs (darunavir, atazanavir, and lopinavir) on specific renal parameters by estimating the predicted time to abnormality. We also defined associated risk factors.

The dose for boosted PIs was standard: atazanavir/ritonavir, 300/100 mg once daily; lopinavir/ritonavir, 400/100 mg twice daily; darunavir/ritonavir 800/100 mg once daily; and nonboosted atazanavir 400 mg once daily. TDF was administered at 300 mg once daily.

The demographic and HIV-related data collected included time on antiretroviral treatment, time on tenofovir, time on PIs, and time on each specific current PI. Blood and urine samples were collected under fasting conditions.

Kidney injury was defined as the presence of micro- or macroalbuminuria or proteinuria, that is, an albumin/creatinine ratio of >30 mg/g and/or protein/creatinine ratio of >200 mg/g and/or the presence of glycosuria (without hyperglycemia), hematuria, and/or hypophosphatemia (<2.5 mg/dL). ^[8]^

Concomitant use of TDF was defined as exposure to the drug for more than 6 months. Patients were considered not to have received TDF if they had never received it, had received it for less than 3 months, or if they had discontinued it for more than 4 months considered the mean time for reversibility of TDF-related toxicity, as is described in a previous study on the reversibility of TDF-related toxicity. ^[9]^

### 2.1 Statistical analyses

Continuous variables were expressed as median (interquartile range), and categorical variables were expressed as frequency (percentage), unless stated otherwise.

Receiver operating characteristic (ROC) curves were used to calculate a cut-off for time on treatment according to the presence or absence of kidney injury. The time for the decision threshold was selected by setting a minimum sensitivity of 80% and the largest specificity available. An area under the curve (AUC) of 0.5 was considered a random allocation; an AUC greater than 0.5 was considered acceptable as a sign of possible relationship.

Statistical significance was set at 0.05.

All candidate predictor variables were entered in a logistic regression analysis, with kidney injury (as defined above) as the dependent variable. The variables included in the logistic regression analysis were time on PIs with and without TDF, time on TDF, time on nucleoside reverse-transcriptase inhibitors, current use of each PI, current use of TDF, age, time since diagnosis of HIV infection (it is defined as the date of the 1st positive HIV test result), time on antiretroviral therapy, age, current and nadir CD4 + cell count, undetectable viral load, hepatitis coinfection, use of nephrotoxic drugs, and body mass index. Variables with a *P*-value <0.20 were entered into the multivariate model. Backward selection (Wald test) was used to build the multivariate model. Forward selection was subsequently applied to verify the stability of the results. Multicolinearity was avoided by excluding from the multiple regression 2 covariates with a correlation coefficient >75%.

All analyses were performed using SPSS (version 15.0; SPSS Inc, Chicago, IL).

## 3 Results

Of the 377 patients included, 104 (29.4%) were receiving lopinavir/ritonavir-containing regimens, with and without TDF, in 28 and 76 cases, respectively; 177 patients (50%) were receiving darunavir/ritonavir, with and without TDF, in 68 and 99 cases; and 73 (20.6%) were receiving atazanavir/ritonavir, with and without TDF, in 30 and 43 cases, respectively. Only 23 patients were receiving nonboosted atazanavir without TDF in all cases.

The demographic and clinical characteristics of the patients and the time on the specific PI are shown in Table 1. Potentially nephrotoxic drugs were collected: angiotensin-converting enzyme inhibitors, angiotensin receptor blockers, cyclosporine, nonsteroidal antiinflammatory drugs, and tacrolimus. After a median (interquartile range) time on the PI of 103 (62–144) months, the percentage of patients fulfilling the criteria for kidney injury (as defined above) was 27.7% for darunavir (27.9% with and 30.3% without TDF), 27.9% for lopinavir (25% with and 29% without TDF), and 30% for atazanavir (20% with and 45% without TDF). No statistically significant differences were observed when we compared each PI with and without TDF. Similarly, no significant differences were observed when we compared all the PIs with TDF and without TDF.

**Table 1:**
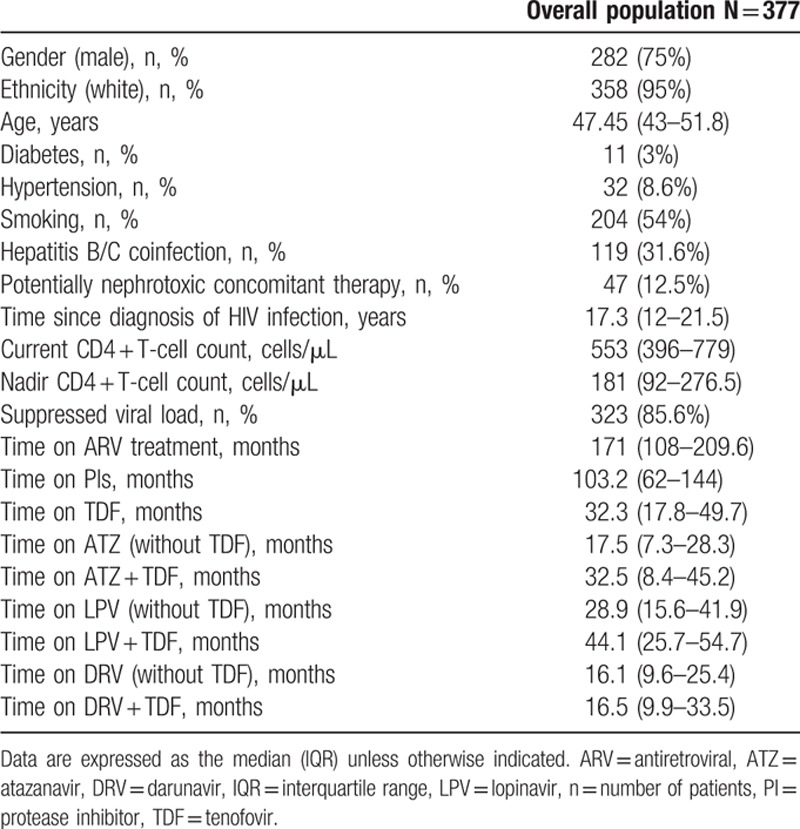
Patient characteristics.

Overall, altered protein/creatinine and/or albumin/creatinine ratios were the most frequent alteration detected (65% of all alterations).

**Figure 1:**
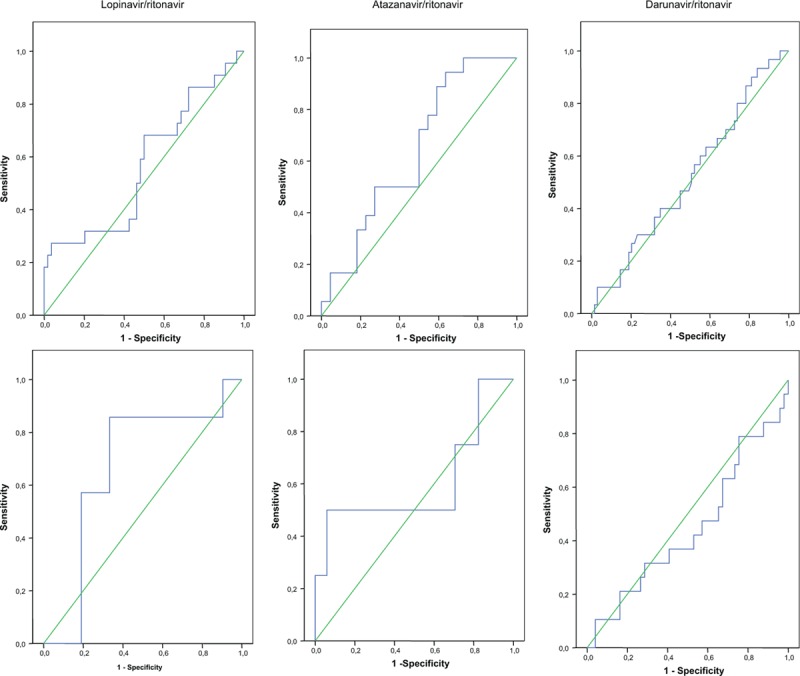
ROC curve is using the top discriminatory features of time on different PIs exposition to classify the individuals as having developed kidney injury or not having developed kidney injury. (A) Boosted PIs without TDF; (B) boosted PIs plus TDF. PI = protease inhibitors, ROC = receiver operating characteristic, TDF = tenofovir).

The ROC curve analysis (Fig. 1) showed an estimated time to kidney injury of 229 days for atazanavir/ritonavir when it was used without TDF (sensitivity, 0.89; specificity, 0.41; and AUC, 0.639), 332 days when it was administered with TDF (sensitivity, 0.75; specificity, 0.29; and AUC, 0.603), and 318 days when nonboosted atazanavir was administered without TDF (sensitivity, 0.89; specificity, 0.29; and AUC, 0.581).

Time until kidney injury was 478 days for lopinavir/ritonavir without TDF (sensitivity, 0.864; specificity, 0.44; and AUC, 0.566) and 1339 days for lopinavir/ritonavir plus TDF (sensitivity, 0.86; specificity, 0.77; and AUC, 0.667).

Estimated time to kidney injury was 283 days for darunavir/ritonavir without TDF (sensitivity, 0.80; specificity, 0.261; and AUC, 0.523) and 286 days for darunavir/ritonavir plus TDF (sensitivity, 0.789; specificity, 0.245; and AUC, 0.446).

The only factor in the logistic regression analysis that was associated with a decrease in the risk of renal abnormalities was current use of lopinavir/ritonavir without TDF (odds ratio = 1.772; 95%CI, 1.070–2.93; *P* = 0.026). No other correlations were established.

## 4 Discussion

We determined predicted time to kidney injury to analyze the impact of various PIs on renal alterations in HIV-infected patients with an eGFR > 60 mL/min/1.73 m^2^. Abundant recent data are available for PIs and renal abnormalities[ ^10^ ^11^] ; however, few data have been reported on the impact of these agents on kidney injury, defined as proteinuria, hematuria, glycosuria, and/or hypophosphatemia. ^[12]^

The importance of an early detection of abnormal biomarkers related with renal damage in the 1st stages, before the advent of clinical symptoms, lies in the possible link with the reversibility of the damage. The safety profile of TDF has been well studied, in contrast, scarce data are available about renal injury with PI strategies.

The 1st remarkable finding of this study is the longer time to kidney injury with lopinavir (478 and 1339 days without and with TDF, respectively). Time to kidney injury was much shorter for the remaining PIs (229 and 332 days for atazanavir and 283 and 286 days for darunavir, without and with TDF, respectively), although the AUC was less accurate in the case of darunavir. These results could reflect lower renal toxicity with lopinavir than with the other PIs, as corroborated by the results of the logistic regression.

Also interesting was the considerably high proportion of patients (30%, similar for all 3 agents) who developed kidney injury, even when TDF was not used concomitantly. This result seems to contradict the results of the logistic regression analysis with respect to lopinavir and the ROC curves with respect to the time taken to develop kidney injury in the case of lopinavir. Nevertheless, compared with the other PIs, exposure to lopinavir was longer, although the number of renal abnormalities was similar to that of the other drugs.

Our results agree with those of previous studies in that the worst result (defined as the shortest time to development of alterations in urinary markers) was with atazanavir/ritonavir, followed by lopinavir/ritonavir.[ ^10^ ^11^ ^13^] Few data are available on the use of darunavir/ritonavir. ^[14]^ Nevertheless, these studies assessed changes in eGFR, incidence of chronic kidney disease, or both, but not the presence of proteinuria or other signs of kidney injury. Atazanavir in particular has the potential to yield crystalline precipitate in urine, leading to crystalluria, tubulointerstitial nephritis, and acute or chronic kidney disease. ^[15]^ In the case of lopinavir/ritonavir, the mechanism that leads to a decline in the eGFR remains unclear, since the results vary depending on the equation used, although ritonavir could play a role in the inhibition of the transport protein of tenofovir. ^[10]^ In addition, lopinavir-related acute interstitial nephritis was recently reported. ^[16]^ Darunavir has only been associated with asymptomatic nephrolithiasis, ^[17]^ although it is the drug with shortest follow-up.

Previous studies confirm increased toxicity of TDF when administered concomitantly with boosted PIs.[ ^4^ ^18^] This effect is related to inhibition of multidrug resistance-associated protein 4 by ritonavir, the subsequent increase in intracellular tenofovir concentrations, and the greater inhibition of DNA-Y polymerase and depletion of mitochondrial DNA.[ ^19^ ^20^] Nevertheless, in our study, time to kidney injury was longer in patients receiving lopinavir or atazanavir concomitantly with TDF. These findings should be interpreted with caution owing to the small sample size in both groups (only 27 and 28 patients with atazanavir and lopinavir, respectively, were also receiving TDF) and to the process for selection of patients, which focused on PIs. The selection criteria excluded patients receiving TDF but not PIs, patients with less than 6 months’ exposure to TDF, patients who discontinued the drug early (3 months), and patients who had interrupted therapy for more than 4 months. The selection process excluded subjects with early toxicity associated with TDF, although it could lead us to underestimate the impact of the drug on kidney markers and thus restrict conclusions about TDF as a separate agent.

Our study has several limitations. Its cross-sectional design could lead us to overestimate the prevalence of kidney injury, because only 1 blood parameter and 1 urine parameter were determined. In addition, no follow-up was available to assess outcome, and no data were recorded for previous antiretroviral history or previous toxicity. Since we included mainly white men, our results cannot be extrapolated to other populations. Statistical methods that include time, such as a Cox model, could not be used owing to the cross-sectional design of the study. Finally, the ROC curve analysis did not show correlations in all cases (e.g., darunavir), probably because the number of patients included or the duration of the evaluation was insufficient.

To conclude, our study identified a considerable proportion of patients with signs of kidney injury while receiving PI-based regimens. The longest time to kidney injury was recorded for lopinavir/ritonavir. Although further longitudinal studies are needed to confirm the long-term consequences of these findings, our results have a key clinical implication: give the high prevalence of renal abnormalities and the recognized silent nature of the initial phases of kidney injury it is important to stress the need to monitor renal parameters, including urine values, even in patients not receiving concomitant tenofovir.

## Acknowledgements

The authors thank Thomas O’Boyle for editorial assistance.
